# GREVE: Genomic Recurrent Event ViEwer to assist the identification of patterns across individual cancer samples

**DOI:** 10.1093/bioinformatics/bts547

**Published:** 2012-09-06

**Authors:** Jean-Baptiste Cazier, Chris C. Holmes, John Broxholme

**Affiliations:** ^1^Wellcome Trust Centre for Human Genetics, University of Oxford, Roosevelt Drive, OX3 7BN and ^2^Department of Statistics, University of Oxford, 1 South Parks Road, OX1 3TG, Oxford, UK

## Abstract

**Summary:** GREVE has been developed to assist with the identification of recurrent genomic aberrations across cancer samples. The exact characterization of such aberrations remains a challenge despite the availability of increasing amount of data, from SNParray to next-generation sequencing. Furthermore, genomic aberrations in cancer are especially difficult to handle because they are, by nature, unique to the patients. However, their recurrence in specific regions of the genome has been shown to reflect their relevance in the development of tumors. GREVE makes use of previously characterized events to identify such regions and focus any further analysis.

**Availability:** GREVE is available through a web interface and open-source application (http://www.well.ox.ac.uk/GREVE).

## 1 INTRODUCTION

Genomic aberrations have been the subject of much interest in the past decade with variable degrees of success. Two categories have to be distinguished: exactly matching germline and unique, often somatic, aberrations. There has been much effort to identify and catalogue the former in order to treat them like regular markers such as SNPs (Iafrate *et al.*, 2004). The first difficulty lies in the exact characterization of the breakpoints. Furthermore, such inventory is impossible for somatic events that are by definition unique. Still, the recurrence of overlapping regions can indicate a key controlling area, e.g. a small deletion on 9p in adolescent acute lymphoblastic leukemia (Paulsson *et al.*, 2008).

Current approaches are essentially based on either the integration into a general-purpose browser to provide context, but no measure of overlap, or the creation of a heatmap where the copy number itself is used as a metric across all types of events to characterize the recurrence (Cancer Genome WorkBench, https://cgwb.nci.nih.gov/cgi-bin/heatmap; Mermel *et al.*, 2011). This single continuous value is then used to construct a score at every location. GREVE is designed to look into further details by allowing the user to define further subgroups such as copy neutral LOH that would be ignored otherwise. Furthermore, GREVE provides a highly configurable interface and specific statistics on recurrent events.

GREVE has been successfully used in numerous cancer studies where the cohort size varied from a handful (Langemeijer *et al.*, 2009; Olsson *et al.*, 2011; Paulsson *et al.*, 2010) to hundreds (Gupta *et al.*, 2008; O’Shea *et al.*, 2009). Highly flexible, GREVE provides the ability to statistically explore a given dataset and to present results in a ready-to-publish format.

## 2 FEATURES

The purpose of GREVE is to enable a flexible view of aberrations across the genome, or per chromosome, and score their recurrence. Therefore, the default usage is very simple with the input of the sole list of events being sufficient, while it is highly configurable with further optional input to help the inspection.

### 2.1 Key features

**Fig. 1.**
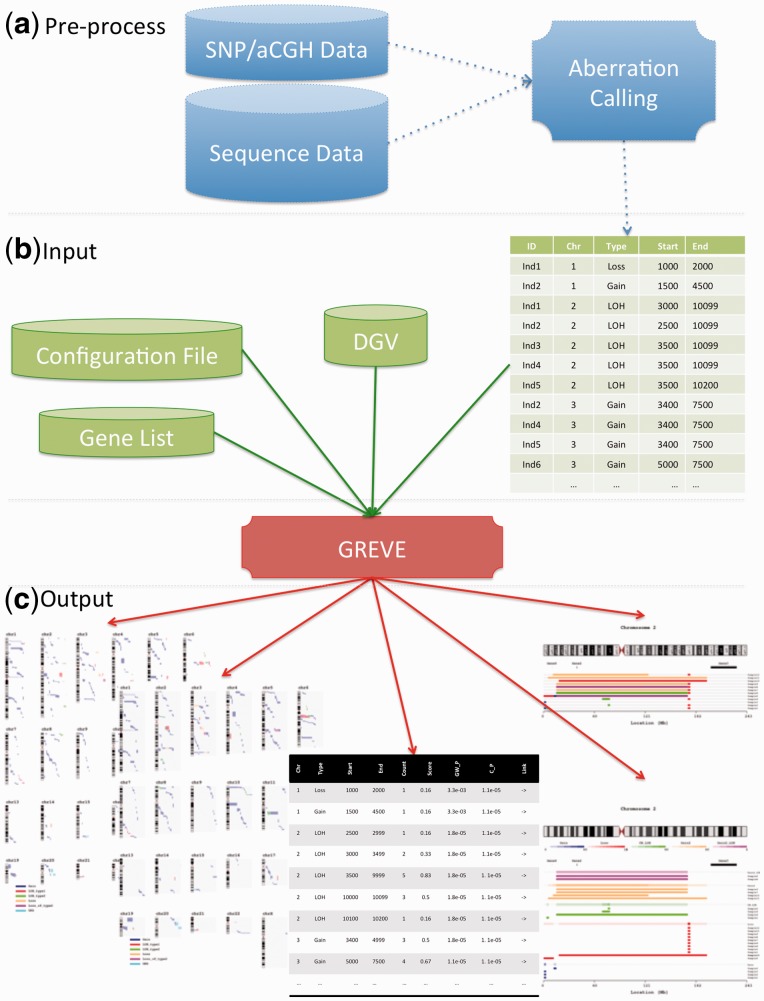
Usage of GREVE where (**a**) pre-processing from any source generates a list of events to be used as (**b**) input together with the optional DGV, Configuration and Gene file. This can generate several output (**c**): two types of genome-wide views (sorted by aberration type or individual), chromosome view with overlap, gene and labels, as well as the detailed list of overlapping events with corresponding counts and statistics

GREVE transforms a list of events into a genomic representation, summarizes and scores their recurrence across samples:

- Read flat or Excel input files containing a list of event per individual and type, with build 35, 36 or 37 location (Fig. 1b).
- Plot all events genome-wide and per chromosome with cytobands. The views can either sort all the events or overlay them with a fixed set of individuals (Fig. 1c).
- Calculate, tabulate and plot recurrence within any given type of events (e.g. Gain, Loss and LOH), score each overlapping segment across multiple statistics (Fig. 1c).
- Generate publication-ready figures in several graphical formats (EPS, PDF, PNG, JPG or TIFF; Fig. 1c).
- Inclusion of key genes on the per-chromosome plot as given in a separate list or known CNV from the Database of Genomic Variants (Iafrate *et al.*, 2004).

### 2.2 Formatting features

Because each study will have varying numbers of individuals and events, the default layout may not be optimal. All positions and colors are available in an optional configuration file.

- Size and color choice for each aberration type.
- Distance between successive events and chromosomes.
- Highlight aberration of certain type (default ‘LOH’).
- Merger of exactly matching events into a larger block.

## 3 IMPLEMENTATION

The GREVE web front end is implemented in HTML/PHP as a wrapper around the Python (van Rossum and Drake, 2001) script engine running on the web server. It requires ImageMagick software (Still, 2005) for figure format conversion from the default Encapsulated PostScript format. The Poisson binomial test is implemented as a wrapper around the *Poibin* R-package (Hong *et al.*, 2011). The web interface allows the upload of all necessary files and a convenient way to select filters and options. It then outputs ready-to-publish figures as well as overlapping details. Examples and frequently asked questions are available on the website.

All the options in the engine software are available through the graphical interface. However, to allow batch processing and further analysis, the source code is available on the website. This should allow specific extensions to match any given project such as subgrouping of individuals (Purdie *et al.*, 2009, 2010). The large demo analysis with 709 events across 30 samples takes 7 s on an AMD64 3.0 GHz processor with 64 Gb of RAM to process with overlaps and scores.

### 3.1 Input files

Only the list of events with corresponding sample labels and type is necessary to run GREVE. It can be generated from the output of various aberration callers from SNP or CGH array as well as sequencing data with eventual post-processing (Fig. 1a and b). Further optional flags, filters and files can be provided to add information or tailor the presentation:

- The list of events with location, sample labels and type of aberration.
- An optional gene list with name and position.
- An optional configuration file allows further tailoring of the figures without the need to modify the program.

### 3.2 Output

The result of the analysis is composed of figures and tables (Fig. 1c):

- Genome-wide and chromosome view of the events in all formats.
- Details of the overlap of events across each chromosome are available directly in the interface as a table or in a separate file.
- The counts and proportion of overlap reflect the comparison to a control set where no somatic event would be expected.
- The Poisson binomial *P*-value tests the probability of a type occurring at the same location depending on the individual proportion on a chromosome (C_P) or genome-wide (GW_P).
